# Continuous Normothermic Machine Perfusion for Renovation of Extended Criteria Donor Livers Without Recooling in Liver Transplantation: A Pilot Experience

**DOI:** 10.3389/fsurg.2021.638090

**Published:** 2021-05-24

**Authors:** Zhitao Chen, Xitao Hong, Shanzhou Huang, Tielong Wang, Yihao Ma, Yiwen Guo, Changjun Huang, Qiang Zhao, Zhiyong Guo, Xiaoshun He, Weiqiang Ju, Maogen Chen

**Affiliations:** ^1^Organ Transplant Center, First Affiliated Hospital of Sun Yat-Sen University, Guangzhou, China; ^2^Guangdong Provincial Key Laboratory of Organ Donation and Transplant Immunology, Guangzhou, China; ^3^Guangdong Provincial International Cooperation Base of Science and Technology (Organ Transplantation), Guangzhou, China; ^4^Department of General Surgery, Guangdong Provincial People's Hospital, Guangdong Academy of Medical Sciences, School of Medicine, South China University of Technology, Guangzhou, China

**Keywords:** continuous normothermic machine perfusion, extended criteria donor liver, early allograft dysfunction, donor after cardiac death, allograft

## Abstract

**Background:** Ischemia injury affects the recovery of liver allograft function. We propose a novel technique aimed at avoiding a second ischemic injury: transplanting an extended criteria donor (ECD) liver directly under normothermic machine perfusion (NMP) without recooling. We studied two cases to evaluate the efficacy and safety of this technique.

**Methods:** The perioperative characteristics and postoperative outcomes of two recipients of ECD livers were analyzed. Both transplantations were performed with continuous normothermic machine perfusion without recooling.

**Result:** In case 1, the cause of donor death was anoxia, and the donor liver had hypernatremia before procurement. The recipient was diagnosed with decompensated cirrhosis. His model for end-stage liver disease (MELD) score was 38. In case 2, the donor liver was from a donor after cardiac death (DCD), and the donor had elevated aspartate aminotransferase (AST) and alanine aminotransferase (ALT) levels. The recipient was diagnosed with acute hepatic failure. His MELD score was 35. Both donor livers were maintained under NMP and then transplanted without recooling. The peak ALT and AST levels after surgery were 452 and 770 U/L in case 1 and 100 and 592 U/L in case 2. Neither early allograft dysfunction (EAD) nor primary graft non-function (PNF) was present in these two cases.

**Conclusion:** In conclusion, our results demonstrate that continuous NMP without recooling is efficacious and safe for LT with extended criteria donor livers. Further investigations of this technique will be performed to confirm these promising results.

## Introduction

Liver transplantation (LT) is a successful treatment option for end-stage liver disease (ESLD) of various etiologies (1). The 1-year survival rate after LT is ~90% thanks to tremendous improvements in surgical methods and immunosuppression (2). Ischemia-reperfusion injury (IRI) constitutes an important cause of PNF, EAD, and biliary complications after LT (3). Normothermic machine perfusion (NMP) is an organ preservation technique that entails continuous or pulsatile circulation of perfusate through the graft and has proven effective in ameliorating IRI (4). In conventional NMP, the liver graft is usually flushed with cold solution before implantation, and IRI is still inevitable (5). We propose a novel technique aimed at avoiding a second ischemic injury by transplanting ECD livers directly under NMP without recooling. We have reported on one patient who underwent this technique, who showed good recovery (6). In this study, we report another two cases to further evaluate the efficacy and safety of this technique.

## Materials and Methods

All the procedures were performed in accordance with the ethical standards of the responsible committee on human institutional and national experimentation and with the Helsinki Declaration of 1964 and its later revisions.

Two patients who had undergone OLT with continuous NMP without recooling were included in the study. The recipient pre-transplantation data and the perioperative data, including the perfusion parameters and postoperative complications, were assessed and analyzed.

### Description of Continuous NMP Without Recooling and Implantation

Details about this technique have been described in previously published articles. Briefly, the donor liver was procured under a standard perfusion technique, and the organ was preserved in a University of Wisconsin (UW) solution prior to perfusion. Afterwards, the donor liver was reprocessed in NMP to 37°C following the flush of lactated Ringer's solution. After ligation of the cystic duct, a tube was placed in it to drain bile. The cannula was placed separately into the infrahepatic inferior vena cava (IHIVC), portal vein (PV) *via* an interposition vascular graft (the donor iliac artery), and the gastroduodenal artery (GDA), respectively, then connected to a liver assist device for perfusion. The perfusate is described in Table 1. Electrolyte balance and acid-base equilibrium were adjusted and maintained throughout the perfusion process. Perfusion was continuous from the time of preservation to the reperfusion.

**Table 1:** The components of the perfusate solution.

**Components**	
Crossed-matched leucocyte-depleted washed red cells	8 U
Gelofusine®	800 ml
5% sodium bicarbonate	150 ml
Heparin	37,500 U
10% calcium gluconate	30 ml
25% magnesium sulfate	2 ml
Methylprednisolone	500 mg
Compound Amino Acid Injection	250 ml
Imipenem cilastatin	0.5 g
Metronidazole	100 ml

**Figure 1:**
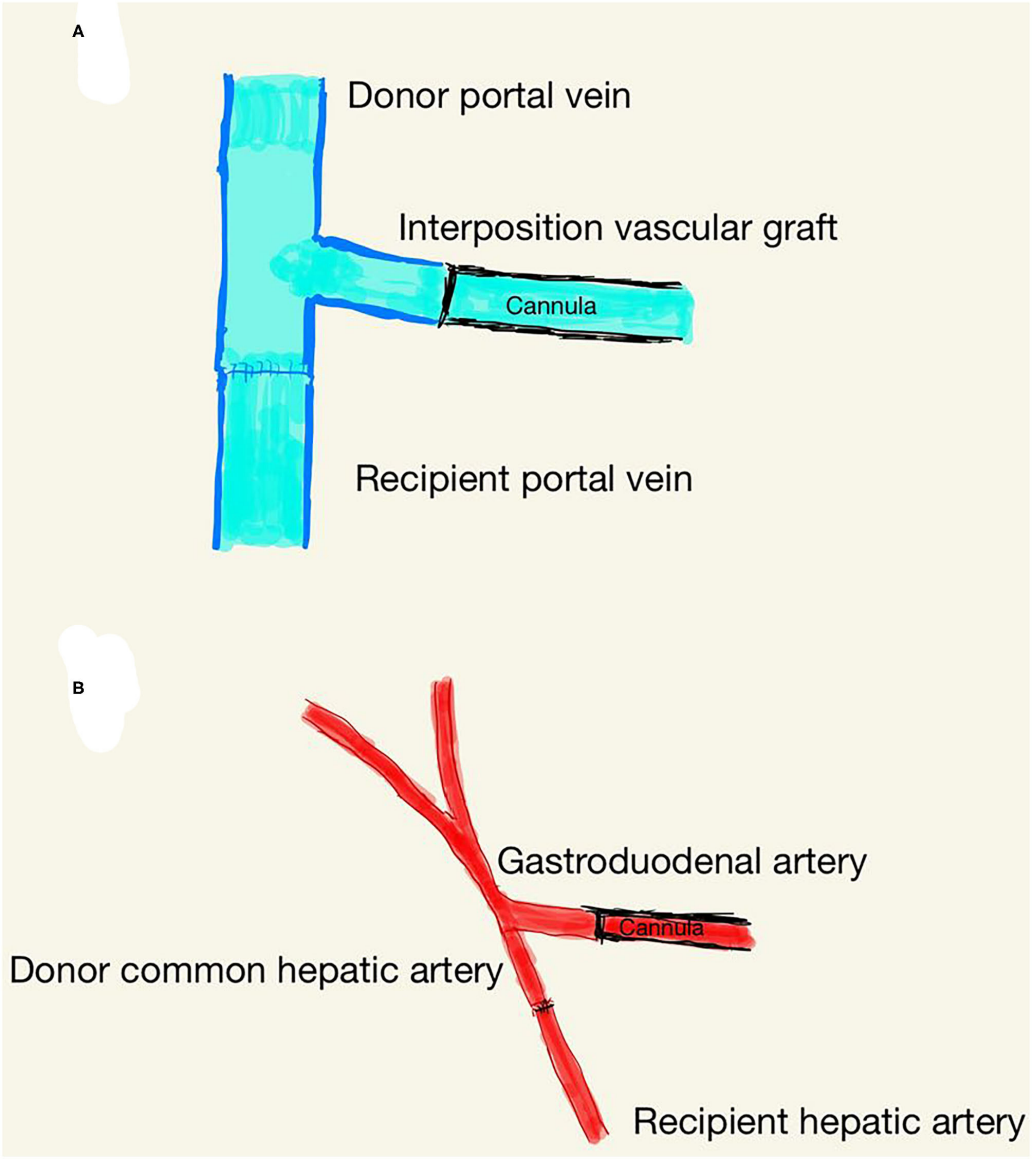
Schematic diagram of portal vein **(A)** and hepatic artery **(B)** anastomosis.

After dissection of the diseased liver, the donor liver was transplanted with an undergoing NMP circuit. We first anastomosed the donor suprahepatic inferior vena cava (SHIVC) and the recipient counterpart in an end-to-end fashion. Subsequently, PV and HA were anastomosed with the donor's counterpart in an end-to-end fashion. During the anastomosis, the blood supply was not stopped. Afterwards, the clamps on the recipient's PV and HA were removed to restore the blood supply to the donor liver soon after NMP was stopped, after removal of the HA and PV cannula (Figure 1). The IHIVC cannula was then removed, and the IHIVC was anastomosed in an end-to-end fashion. After removal of the drainage tube, the common bile duct was anastomosed end-to-end.

### Posttransplant Care

After the transplantation, all patients were transferred to the intensive care unit with the endotracheal tube. After the tubes were removed, the patients were transferred to the general ward for further care. Biomedical values including regular blood test, transaminase, and bilirubin were tested and documented every day in the first week. Routine Doppler ultrasound of the liver graft blood flow and biliary tract was performed once every 2 days for 7 days. The immunosuppressive regimen was similar for all patients and included tacrolimus and mycophenolate mofetil. Basiliximab (20 mg) was administered intraoperatively before closing the abdomen and the 4th day postoperation by intravenous pump for induction immunotherapy. The blood levels of tacrolimus were 8–12 ng/ml in the first month, followed by 4–8 ng/ml thereafter.

## Results

Data of these two cases are presented in Table 2. In case 1, the serum AST, ALT, and total bilirubin (Tbil) levels of the donor before procurement were 56.8 U/L, 26.2 U/L, and 10.7 μmol/L, respectively. Hypernatremia (sodium, 165 mmol/L), anoxia as the cause of death, and a long cold ischemia time of more than 8 h were the risk factors for failed liver transplantation. In case 2, the AST, ALT, and TBil levels of the donor were 163 U/L, 151 U/L, and 6.2 μmol/L, respectively. Risk factors were as follows: donor after cardiac death (DCD), anoxia as the cause of death, and a cold ischemia time of more than 10 h. All risk factors made these two donors ECDs.

**Table 2:** Perioperative data of 2 patients with continuous NMP without re-cooling.

	**Donor 1**	**Donor 2**
Donor type	DBD	DCD
Sodium (mmol/L)	165	150
Potassium (mmol/L)	3.66	3.50
Hemoglobin (g/L)	114	79
AST(U/L)	56.8	163
ALT(U/L)	26.2	151
GGT(U/L)	27	31
Bilirubin (mmol/L)	10.7	6.2
Anhepatic time, min	86	51
CIT, h	8	10
WIT, min	0	8
	**Patient 1**	**Patient 2**
MELD	38	35
Preoperative AST(U/L)	11	50
Preoperative ALT(U/L)	39	35
Preoperative Bilirubin (mmol/L)	152.8	440.8
Total operation time (mins)	470	460
Anhepatic time (mins)	55	51
Intraoperative transfusions (U)	26	20
Blood loss (ml)	6,000	4,000
ICU length-of-stay (h)	105	216
Peak AST(U/L)	770	592
Peak ALT(U/L)	452	100
INR on POD 7	1.35	1.23
Bilirubin (mmol/L) on POD7	86.8	78.9
EAD	0	0
PNF	0	0
Biliary anastomotic strictures	0	0
Hepatic artery complications	0	0
Acute kidney injury	0	1

*DBD, Donor after brain death; DCD, Donor after cardiac death; AST, Aspartate aminotransferase; ALT, Alanine aminotransferase; GGT, γ-glutamyl transpeptidase; CIT, Cold ischemia time; WIT, Warm ischemia time; MELD, Model for end-stage liver diseases; ICU, Intensive care unit; INR, International normalized ratios; EAD, Early allograft dysfunction; PNF, Primary nonfunction; POD, postoperative day*.

The recipient patient in case 1 was diagnosed with hepatitis B virus-associated (HBV) decompensated cirrhosis. He had a history of esophageal and gastric variceal bleeding and abdominal bleeding. His serum AST, ALT, and Tbil levels were 11 U/L, 39 U/L, and 152.8 μmol/L, respectively. His MELD score was 38. The recipient in case 2 was diagnosed with acute hepatic failure and hepatic encephalopathy. He had a history of cerebral infarction, hypertension, and HBV infection. His AST, ALT, and Tbil levels were 50 U/L, 35 U/L, and 440.8 μmol/L, respectively. His MELD score was 38.

**Figure 2:**
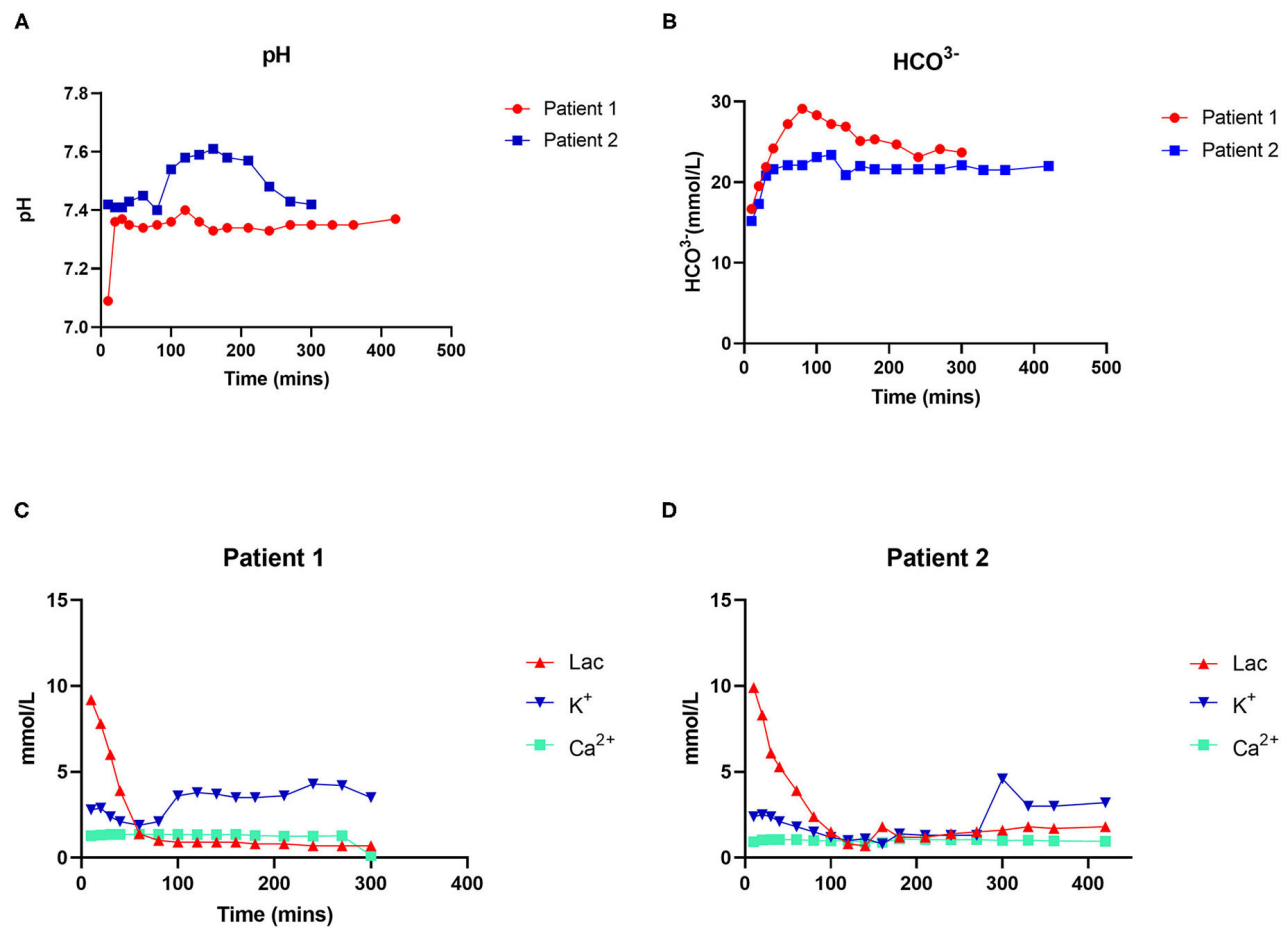
Dynamic changes in physicochemical indexes during perfusion. **(A)** Changes in pH. **(B)** Changes in HCO^3−^. **(C)** Changes in lactate and ion concentrations in patient 1. **(D)** Changes in lactate and ion concentrations in patient 2.

NMP was performed for a total of 7 h for each donor liver (Figure 2). During the perfusion process, the electrolyte balance and acid-base equilibrium were adjusted and maintained. The pH during perfusion was adjusted and maintained at 7.37 and 7.42 in cases 1 and 2, respectively. The lactate concentration dropped significantly, from 9.9 to 2.4 mmol/L after 80 min of perfusion in case 1 and from 9.2 to 1.4 mmol/L after 80 min of perfusion in case 2. The concentrations of calcium and sodium remained stable in both cases. The anhepatic time was 55 and 51 min in cases 1 and 2, respectively.

**Figure 3:**
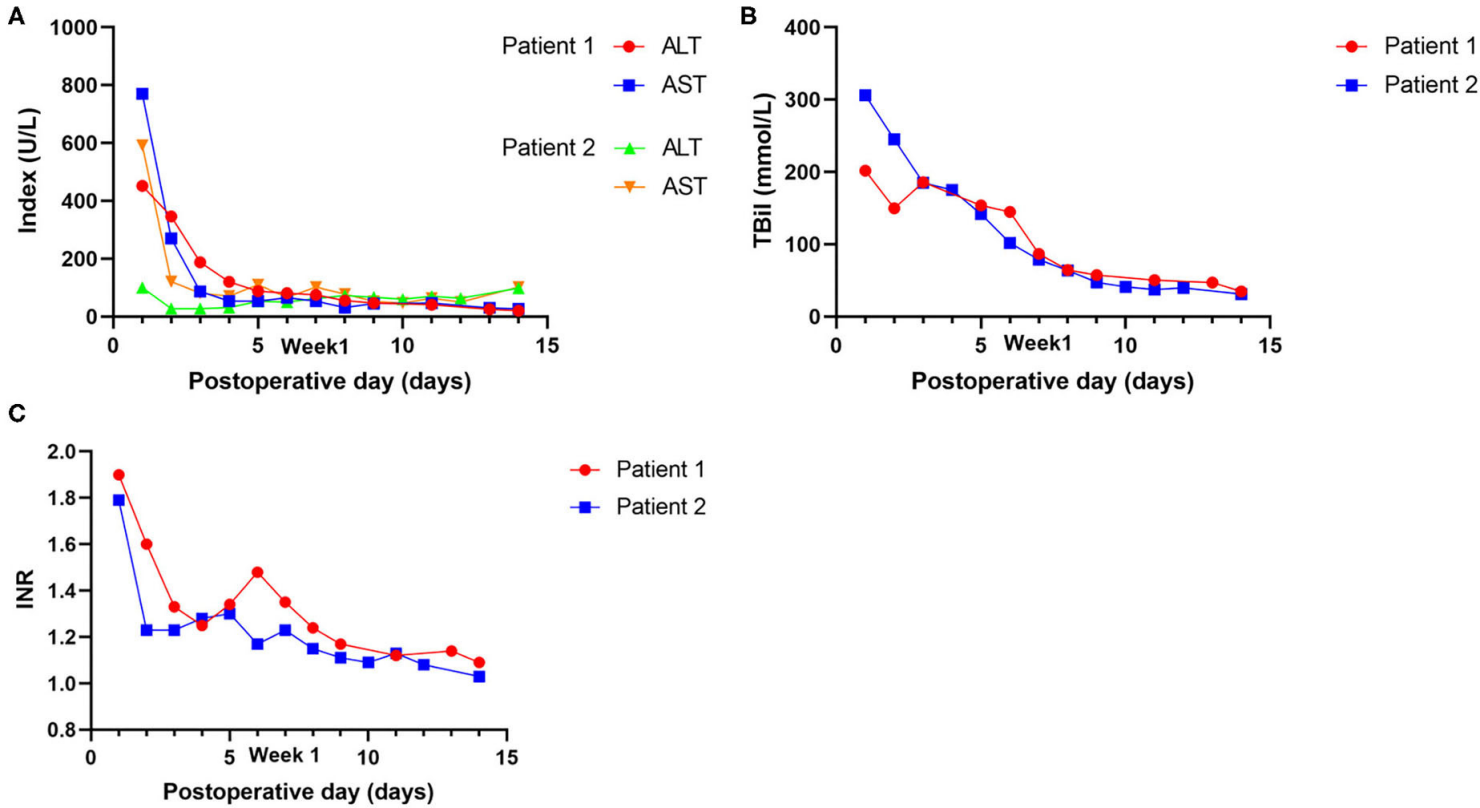
Postoperative outcomes of laboratory tests of the recipients. **(A)** Changes in transaminases (AST and ALT). **(B)** Changes in Tbil. **(C)** Changes in INR.

Postoperative data are presented in Table 2. The intensive care unit (ICU) stay times were 105 and 216 h, respectively. The maximal ALT and AST were 452 and 770 U/L in case 1 and 100 and 592 U/L in case 2, respectively. The international normalized ratio (INR) and TBil on postoperative day (POD) 7 were 1.35 and 86.8 mmol/L in case 1, respectively, and 1.23 and 78.9 mmol/L in case 2 (Figure 3). Neither EAD nor PNF was presented in either case. Patient 2 suffered from acute kidney injury (AKI).

## Discussion

In conventional LT, cryopreservation can reduce the physiological metabolism of cells but cannot slow down the damage to cell integrity (7). IRI represents one of the major challenges during liver surgery and LT. To minimize the risk of PNF or EAD caused by IRI, we designed a novel method called continuous NMP without recooling for ECD livers. A case report on the feasibility of avoiding cooling was authored by Fabrizio et al. (8). However, they do not describe the details of the specific procedures. Ju et al. were the first to describe how to avoid a second cooling before implantation and proved that doing so helps reduce the risk of PNF or EAD (6). In this study, we report two cases of LT following this technique.

ECD livers are thought to be of lower-than-average quality and to be associated with poorer postoperative outcomes (9). Maring et al. suggested that ECD livers are associated with older donor age, longer warm ischemia time, moderate or severe microvascular steatosis, and HBsAg positivity (10). The review by Vodkin et al. noted that ECD livers are correlated with older donor age, steatosis, DCD, and donors with an increased risk of disease transmission (11). In our study, both donor livers had several risk factors, confirming that they were ECD livers. They were not suited for transplantation with conventional cold preservation. Tchilikidi et al. suggested that ECD transplantation required measures for graft preservation and assessment (12). NMP provides an effective method for salvaging ECD organs. Davide et al. retrospectively studied 34 transplantations of DCD donors using NMP and showed that it would be feasible for DCD LT (13). The prospective clinical trial of Otto et al. reported that NMP was a safe option for risky donor livers and increased the number of available donor livers (14). Nevertheless, current NMP techniques have potential problems and need innovation. Our novel technique is designed to solve this problem: during preservation and implantation, the blood supply is not stopped. In our cases, the laboratory test results fell from above the normal ranges to within the normal ranges within 1 week. Neither EAD nor PNF was presented. However, further randomized clinical trial (RCT) data are needed to confirm its safety and efficacy.

The limitations of our study are as follows. First, the preservation (the establishment of portal interposition vascular graft and the connection of cannulas) and the implantation are complex and need to be further modified. In addition, its effectiveness needs to be proven in a RCT.

In conclusion, our results demonstrate that continuous NMP without recooling is efficacious and safe for LT with extended criteria donor livers.

## Data Availability Statement

The original contributions presented in the study are included in the article/supplementary material, further inquiries can be directed to the corresponding author/s.

## Ethics Statement

The studies involving human participants were reviewed and approved by Institutional Ethics Committee for Clinical Research and Animal Trials of the First Affiliated Hospital of Sun Yat-sen University. Written informed consent for participation was not required for this study in accordance with the national legislation and the institutional requirements.

## Author Contributions

WJ and MC: conceptualization. ZG and XHe: methodology. ZC, XHo, TW, and SH: writing–original draft. YM, CH, YG, and QZ: data collection. MC: writing—review and editing. WJ: supervision. XHe: project administration. WJ, MC, and XHe: funding acquisition. All authors contributed to the article and approved the submitted version.

## Conflict of Interest

The authors declare that the research was conducted in the absence of any commercial or financial relationships that could be construed as a potential conflict of interest.

## Abbreviations

AKI: acute kidney injury
AST: aspartate transaminase
ALT: alanine transaminase
DBD: donor after brain death
DCD: donor after cardiac death
EAD: early allograft dysfunction
ECD: extended criteria donor
ESLD: end-stage liver disease
GDA: gastroduodenal artery
HBV: hepatitis B virus
HA: hepatic artery
ICU: intensive care unit
IRI: ischemia-reperfusion injury
INR: international normalized ratio
IHIVC: infrahepatic inferior vena cava
LT: liver transplantation
MELD: model for end-stage liver disease
NMP: normothermic machine perfusion
PV: portal vein
PNF: primary graft non-function
POD: postoperative day
RCT: randomized clinical trials
SHIVC: suprahepatic inferior vena cava
Tbil: total bilirubin
UW: University of Wisconsin.
